# Pernicious Anemia: The Hematological Presentation of a Multifaceted Disorder Caused by Cobalamin Deficiency

**DOI:** 10.3390/nu14081672

**Published:** 2022-04-17

**Authors:** Gianluca Esposito, Ludovica Dottori, Giulia Pivetta, Irene Ligato, Emanuele Dilaghi, Edith Lahner

**Affiliations:** Medical-Surgical Department of Clinical Sciences and Translational Medicine, Sant’Andrea Hospital, University Sapienza, 00189 Rome, Italy; gianluca.esposito@uniroma1.it (G.E.); ludovica.dottori@uniroma1.it (L.D.); giulia_pivetta@libero.it (G.P.); irene.ligato@uniroma1.it (I.L.); emanuele.dilaghi@uniroma1.it (E.D.)

**Keywords:** atrophic gastritis, autoimmune gastritis, blue light imaging, cobalamin, surveillance, gastric cancer, gastric neuroendocrine tumor, intestinal metaplasia, iron deficiency anemia, narrow-band-imaging endoscopy, pernicious anemia

## Abstract

Pernicious anemia is still a neglected disorder in many medical contexts and is underdiagnosed in many patients. Pernicious anemia is linked to but different from autoimmune gastritis. Pernicious anemia occurs in a later stage of autoimmune atrophic gastritis when gastric intrinsic factor deficiency and consequent vitamin B_12_ deficiency may occur. The multifaceted nature of pernicious anemia is related to the important role of cobalamin, which, when deficient, may lead to several dysfunctions, and thus, the proteiform clinical presentations of pernicious anemia. Indeed, pernicious anemia may lead to potentially serious long-term complications related to micronutrient deficiencies and their consequences and the development of gastric cancer and type 1 gastric neuroendocrine tumors. When not recognized in a timely manner or when pernicious anemia is diagnosed with delay, these complications may be potentially life-threatening and sometimes irreversible. The current review aimed to focus on epidemiology, pathogenesis, and clinical presentations of pernicious anemia in an attempt to look beyond borders of medical specialties. It aimed to focus on micronutrient deficiencies besides the well-known vitamin B_12_ deficiency, the diagnostic approach for pernicious anemia, its long-term complications and optimal clinical management, and endoscopic surveillance of patients with pernicious anemia.

## 1. Introduction

Pernicious anemia (PA) is an autoimmune disease, probably much more common as currently diagnosed, that is still neglected in many medical contexts and underdiagnosed in many patients.

PA is linked to autoimmune gastritis (AAG), but PA and AAG are not synonyms, as PA occurs in a later stage of AAG when gastric intrinsic factor deficiency and consequent vitamin B_12_ deficiency may occur.

The multifaceted nature of PA is related to the important role of cobalamin, which when deficient, may lead to several dysfunctions ranging from hematopoiesis to neurological, psychiatric, and obstetric abnormalities. The pathogenesis of PA has not been clarified, but it is likely linked to the autoimmune destruction of gastric glands due to autoreactive T lymphocytes in genetically predisposed individuals. The role of previous *Helicobacter pylori* infection as a supposed but not yet definitely proven trigger of gastric autoimmunity cannot be excluded.

An important point is that PA may lead to potentially serious long-term complications that may be related to micronutrient deficiencies and the development of gastric neoplasms, in particular, gastric cancer and type 1 gastric neuroendocrine tumors. When not recognized in a timely manner or when PA is diagnosed with delay, these complications may be potentially life-threatening and sometimes irreversible.

The current review aimed to focus on the epidemiology, the pathogenesis, and the clinical presentations of PA in an attempt to look beyond borders of medical specialties. Further, it aimed to focus on micronutrients deficiencies and malabsorption besides the well-known vitamin B_12_ deficiency. Finally, it aimed to focus on the diagnostic approach for PA, ranging from biochemical tests to high-quality endoscopy and histopathology, as well as on the long-term complications and the optimal clinical management and endoscopic surveillance of patients with PA.

## 2. Epidemiology, Pathogenesis, and Clinical Presentations of Pernicious Anemia: Looking beyond Borders of Medical Specialties

### 2.1. Epidemiology of Pernicious Anemia: A Not Completely Investigated Issue

PA is a macrocytic anemia due to vitamin B_12_ (cobalamin) malabsorption as a consequence of intrinsic factor deficiency [1]. Generally, it takes about 10–12 years to clinically develop symptomatic PA, so PA may onset with subclinical vitamin B_12_ deficiency [2]. In PA, the underlying pathogenetic mechanism is AAG, an organ-specific immune-mediated disorder featuring the damage of the gastric parietal cells involved in the secretion of intrinsic factor and hydrochloric acid by the gastric proton pump [3]. The presence of anti-parietal cell antibodies (PCAs) directed towards the gastric proton pump (gastric H^+^/K^+^ ATPase) as well as antibodies against intrinsic factor (IF) (although in a lower percentage) are commonly associated with gastric corpus atrophy and intrinsic factor deficiency. Atrophic corpus gastritis is a chronic disease defined as a decrease in or loss of the original gastric glands, replaced by pseudo-pyloric or intestinal metaplasia or fibrosis [4]. Gastric corpus atrophy is a necessary but insufficient condition for the onset of PA, as gastric corpus atrophy may also take its course without PA. PA has often been confused with vitamin B_12_ deficiency (actually, PA denotes only vitamin B_12_ deficiency due to gastric atrophy and/or intrinsic factor deficiency) or AAG impairing epidemiological data. PA is considered a late stage of AAG.

Epidemiological studies have indicated that PA affects 0.1% of the general population and 2–3% of subjects aged >65 years (female:male ratio ~2:1) [3]. PA may affect people of all ages, but its incidence typically increases with age. In the latest Italian and Turkish studies, the average age of men affected by PA was approximately 49–55 years, compared with 40–61 for women [4,5]. Similarly, in a prospective survey of cobalamin status in the elderly that included 729 subjects (≥60 years old), the overall frequency of previously undiagnosed PA was about 2%, being higher in black (4.3%) and white women (4.0%) [6]. Likewise, even if PA typically affects elderly women of Northern European ethnicity, recent studies have shown an overlapping prevalence of PA in other ethnicities (e.g., Caucasian, African, American), with an earlier onset of the pathological condition [7].

Furthermore, PA associated with common variable immunodeficiency, low serum immunoglobulin concentrations, selective IgA deficiency, or the childhood PA, should be distinguished from classic PA due to both an earlier onset of symptoms and the absence of an association with gastric corpus atrophy of the formers. In particular, childhood PA is characterized by either a genetic inability to secrete intrinsic factor or secretion of a defective intrinsic factor [8]. However, these particular forms are very rare.

From a clinical perspective, the fact that PA often develops without symptoms leads to underestimating the real prevalence of PA diagnoses and complications. A recent cross-sectional study on upper gastrointestinal symptoms in AAG involving 379 symptomatic participants with AAG revealed the presence of PA in 53.6% of cases [9]. A systematic review of PA complications highlighted an overall cumulative incidence of cancer of 2.4% and a gastric cancer incidence of 0.3/100 person-years [10]. Concerning vitamin B_12_, by referring to the National Health and Nutrition Examination Survey data, and considering serum vitamin B_12_ cut-off values (<148, <200 and <256 pmol per liter), the prevalence of low levels of vitamin B_12_ was estimated to be 2.9%, 10.6%, or 25.7%, respectively; however, this survey is not limited to PA but extended to all causes of vitamin B_12_ deficiency [11]. Other studies showed that prototypical socio-demographical factors contribute to B_12_ blood levels of the population. The incidence of PA was found to be lower in Africans (although there is a prevalent deficiency of vitamin B_12_ due to insufficient dietary intake) and higher in North European citizens (due to the greater prevalence of autoimmune diseases) [12].

Concerning chronic atrophic gastritis, the worldwide prevalence in the general population was reported to be 23.9% according to serology tests and 33.4% using biopsies [13]. Similarly, while serology tests identify a prevalence of AAG of 8–20%, studies reporting histological results estimate a prevalence of about 0.5–4.5% [14]. Likewise, AAG often also develops without symptoms in the early stages, so is it likely being underestimated by epidemiological studies.

To date, it is still difficult to draw coherent conclusions of the real occurrence, prevalence, incidence, and features of PA, given the scarcity of available reports, several methodological differences, and inconsistencies in the results. Large multicenter high-quality studies are needed to gain a global and more truthful overview of the real frequency and associated features of PA. Table 1 summarize the main epidemiological data.

### 2.2. Pathogenesis of Pernicious Anemia: More Than One Player on the Field

In PA, gastric oxyntic mucosa is destroyed with the loss of parietal cells, resulting in IF and hydrochloric acid (HCl) deficiency, thus impairing absorption of vitamin B_12_ and other micronutrients (see paragraph 3 below). Typically, PA is associated with the presence of autoantibodies against IF (IFA) and parietal cells (PCA), thus supporting the autoimmune origin of this condition [15]. PA is considered the end-stage of AAG, frequently presenting with other autoimmune conditions such as autoimmune thyroid disease, type 1 diabetes, and vitiligo [8]. Another peculiar feature of AAG is corpus-restricted atrophy and the absence of *Helicobacter pylori* (*H. pylori*) at histology. IFA are class G immunoglobulins that target the binding site for cobalamin (type I) or the binding site for ileal epithelial mucosa (type II) [16]. PCA are class G immunoglobulins directed towards the α and β subunits of the gastric proton pump (H⁺/K⁺ ATP-ase) [17,18]. These autoantibodies are released from plasma cells activated by autoreactive CD4+ T lymphocytes [19].

AAG (corpus-restricted atrophy) and *H. pylori* atrophic multifocal (antrum-involved) gastritis are commonly considered two distinct entities, as stated by the currently used updated Sydney System which distinguishes autoimmune atrophic gastritis (formerly type A gastritis) and multifocal atrophic gastritis (formerly type B gastritis) [20]. In recent years, it has been observed that long-standing *H. pylori* infection can be implicated in the pathogenesis of AAG and in the induction of gastric autoimmunity [21,22,23]. *H. pylori*-induced antibodies have been reported to cross-react with human gastric mucosa [24], probably due to molecular mimicry between H⁺/K⁺ ATP-ase subunits and *H. pylori* antigens [25,26,27,28], even more so in genetically susceptible individuals [29]. Furthermore, the concomitant autoimmune disorders and the presence of autoimmunity-related HLA patterns had a similar frequency in corpus-restricted and multifocal atrophic gastritis, showing that these two entities may be overlapping and may share some pathogenetic pathways [30,31]. The overlap between autoimmune and *H. pylori* atrophic gastritis is also highlighted by the evidence of positivity to *H. pylori* (histology, ELISA and immunoblotting serology) in patients with AAG [32,33,34,35], and on the other hand, the presence of PCA positivity (considered as the hallmark of autoimmune AAG) [24,36] in *H. pylori*-related atrophic and non-atrophic gastritis [36]. In a series of patients with PA, 60.5% were found to be *H. pylori-*positive, 50.6% had only positive anti-*H. pylori* IgG serology, but 9.9% had positive histology [35]. A case–control study showed a similar frequency of PCA positivity assessed with ELISA in patients with multifocal atrophic gastritis and in those with corpus-restricted atrophic gastritis (=AAG) [37], suggesting that the presence of PCA seems not to be an exclusive feature of AAG, but an expression of the damaged oxyntic mucosa, present also in *H. pylori*-infected atrophic gastritis patients. According to these data, it seems reasonable to assert that long-standing *H. pylori* infection may induce an inflammatory response that, especially in genetically susceptible patients, can trigger the autoimmune process, finally causing irreversible destruction of gastric oxyntic mucosa, hypo-achlorhydria, and lack of IF. In this scenario, the presence of *H. pylori* may not be necessary once the autoimmune process has been turned on [38].

In PA, genetic susceptibility for autoimmunity may also play a role, as seen for other autoimmune diseases [39]. In particular, HLA-DRB1*03 and HLA-DRB1*04, known for their implication in autoimmune thyroiditis and type 1 diabetes [40], were found to be significantly associated with PA [30], supporting its autoimmune origin. Recently, the role of genetic predisposition in developing and manifesting PA has been analyzed. A further implication of HLA alleles was found, and HLA-DRB1*0404, HLA-DQA1*0301, DQB1*0302, and DRB1*0201 were associated with the presence of autoantibodies against the subunit A of the proton pump, together with some variants of PTPN22 and CTLA4, both involved in the immune response [41]. A genome-wide association study published in 2021 identified four risk loci for PA in or near candidate genes for autoimmune diseases (AIRE, HLA, PTPN22 and IL2RA), and suggested PNPT1 [42], a polyribonucleotides nucelotidyltransferase that participates in mitochondrial RNA transfer and whose dysfunction leads to immune activation [43]. Genetic variants may also have a role in the clinical manifestation of B_12_ deficiency, as shown in a study examining the presence of putative single-nucleotide polymorphisms (SNPs) related to vitamin B_12_ levels in patients with PA, which found out that a genetic variant of TCN2 (rs9606756) encoding for transcobalamin 2 occurred more frequently in patients with PA that in controls [44]. Thus, in PA, more than one player is on the field whose specific roles and mutual interactions still await definite clarification.

### 2.3. Clinical Presentation of PA: Many Faces, One Story

Clinical manifestations of PA are variegated, often insidious, and may involve many organs and systems, necessitating a global medical approach for its identification and management.

The main presentations of PA are hematological and neurological consequences of vitamin B_12_ deficiency, and both require several years for their development. Vitamin B_12_ deficiency impairs hemopoiesis due to the pivotal role of vitamin B_12_ in DNA synthesis. Macrocytic anemia with MCV ≥ 100 fL is the hallmark of PA. Nevertheless, this feature is not always present at diagnosis, as almost 30% of patients do not present macrocytosis [45] but normocytic anemia. This typically occurs in the case of concomitant iron deficiency and/or other diseases causing microcytosis. Conversely, macrocytosis is often the first presentation of PA for months or years before anemia sets up. While anemic patients may present symptoms related to anemia itself, such as weakness, reduced mental concentration, headache, palpitations, or, rarely, cardiological chest pain, patients presenting with non-anemic macrocytosis may present with neurological symptoms [46]. Other important hematological manifestations of PA and vitamin B_12_ deficiency are hyperhomocysteinemia-related thrombosis [47,48,49] and bone marrow failure with pancytopenia, whose differentiation from other causes of bone marrow failure may be challenging since morphological changes in hematopoietic cells and the bone marrow are often overlapping and not characteristic for PA [46,50].

Neurological manifestations are caused by demyelination induced by vitamin B_12_ deficiency [51], whose peak expression is subacute combined degeneration, characterized by lesion of the posterior and lateral columns of the spinal cord leading to asthenia, spasticity, impaired vibratory and proprioceptive sensation with ataxia and extensor plantar responses [52]. The most common initial manifestation of vitamin B_12_ deficiency is paresthesia, present in 70% of patients with neurological symptoms [45]. Paresthesias are described as tingling or numbness, and, in contrast with other neuropathies, typically start in hands or both distal extremities [45,52]. Other neurological manifestations may include autonomic dysfunction (erectile and bladder dysfunction) [53], optic neuropathy with progression to visual loss (characterized by central and centrocecal scotomas) [54], and memory and mood involvement, up to dementia.

Neuropsychiatric syndromes may also be present in patients without hematological signs, and can be caused by both derangement in monoamine neurotransmitter production and secondary increase in homocysteine and methylmalonic acid [55]. Significant manifestations are mood disorders, such as depression and mania, chronic fatigue syndrome, and psychosis [56]. Psychosis may present with suspiciousness, disorganized thought processes, and auditory and visual hallucinations [57].

Cognitive impairment has been extensively associated with vitamin B_12_ deficiency, manifesting with memory impairment, slow mentation, attention deficit, and dementia [58,59]. Low serum B_12_ was shown to be associated with Alzheimer’s disease, vascular dementia, and Parkinson’s disease, as reported by several authors; this association is reported also for low–normal levels of B_12_ (≤250 pg/L), and a small subgroup of dementias are responsive to vitamin B_12_ supplementation [60].

Neurological symptoms may be reversible with vitamin B_12_ treatment if recognized in a timely manner. Therefore, it is important to pay attention and intercept initial signs before neurological lesions become irreversible [46]. In this context, it should be kept in mind that copper deficiency may mimic clinical/hematological presentation of vitamin B_12_ deficiency and should be accurately excluded, in particular in non-responders to cobalamin supplementation [61,62].

Gastrointestinal manifestations involve symptoms related to the condition of AAG, which PA arises from. The most frequent symptoms are all included in the wide spectrum of dyspepsia, such as epigastric pain (35.5%), early satiety (10%), and postprandial bloating and fullness (7%) [9,63]. These symptoms may be directly correlated to hypochlorhydria which can induce itself impaired gastric emptying [64], while duodenal alterations, more recently linked to dyspepsia, remain to be elucidated in PA/AAG [65].

In a small portion of patients with AAG/PA, reflux-like symptoms may be present, probably due to nonacidic refluxes that may play a role in the heartburn perception [66]. A cross-sectional study on gastrointestinal symptoms in AAG also reported a less-frequent association with lower-GI symptoms such as functional abdominal pain syndrome, irritable bowel syndrome, functional diarrhea, and functional bloating [9].

Clinical manifestations of PA can involve signs other than typical hematological and neurological ones. A look must be taken at the presence of other autoimmune diseases, especially autoimmune thyroid disease, type 1 diabetes, and vitiligo. Autoimmune thyroid disease is reported in up to 40% of AAG patients [67]; in patients with concomitant autoimmune thyroid disease and PA, physicians should be aware of the possible need for an increased dose of replacement drug due to an impairment in thyroxin absorption induced by hypochlorhydria [68].

The association with other autoimmune diseases has been less investigated, but links with celiac disease [63,69], hyperparathyroidism [70], rheumatoid arthritis [63], myasthenia gravis [71], inflammatory bowel disease (76), systematic lupus erythematosus [72] and autoimmune liver disease [73] have been reported.

It is important to keep in mind that other conditions, less frequently associated with PA, might be presentations of B_12_ insufficiency or deficiency, such as infertility, birth defects, orthostatic hypotension, skin change, or glossitis [74].

Moreover, the interlink between PA, vitamin B_12_ deficiency, and hyperhomocysteinemia should not be forgotten, because of its implication in cardiovascular diseases. Hyperhomocysteinemia is considered a risk factor for cardiovascular diseases; it is linked to occlusive artery disease, especially brain lesion stroke, in the heart, in the kidney, and in addition to venous thrombosis [75,76].

## 3. Micronutrients’ Malabsorption and Deficiencies in Pernicious Anemia: The Known and the Forgotten

It is well-known that the stomach plays an important role in the homeostasis of the hematopoietic vitamin B_12_, but this role for the other pivotal hematopoietic iron is less diffuse, and even less so is the role of the stomach in the homeostasis of other, similarly important micronutrients, such as ascorbic acid and calcium [77,78,79].

### 3.1. Hypochlorhydria, the Central Player in Pernicious Anemia and Micronutrients’ Malabsorption

The pathogenetic basis of PA is recognized in AAG, in which the proximal part of the stomach (fundus and corpus) is affected, characterized by oxyntic glands including parietal cells whose main role is hydrochloric acid secretion and IF production [80]. The proton pumps (H^+^, K^+^ ATPase) produce HCl, and its secretion is coordinated and regulated by several signals; in particular, histamine, gastrin, and acetylcholine have a stimulating action, even toward IF secretion [81,82,83].

An autoimmune injury due to autoreactive T cells and the consequent production of PCA and IFA lead to atrophy of the gastric glands and give rise to loss of functioning of these highly specialized cells. In this situation, gastric acid secretion is importantly reduced and intragastric pH increased, leading to hypochlorhydria. As promptly explained in the following paragraphs, hypochlorhydria can be considered as the starting point for many pathways in PA that lead to micronutrients’ malabsorption and deficiencies.

### 3.2. Vitamin B_12_, the Well-Known Factor Unaccounted for in Pernicious Anemia

Vitamin B_12_ is mainly acquired from animal products, such as meat, fish, eggs, and dairy products. The daily necessary amount of B_12_ is 2–3 μg and a large part of this is stored in the liver; so, generally, it takes years before cobalamin deficiency develops and manifests itself [11,84]. Cobalamin absorption and subsequent utilization require exact steps, with the stomach playing a central role. Briefly, gastric acidity and pepsin induce cobalamin proteolysis from food proteins, and subsequently, cobalamin is bonded with haptocorrin produced by salivary glands [85]. In the duodenum, pH changes induce cobalamin dissociation from haptocorrin and its ligation with intrinsic factor, which is produced by parietal gastric cells; IF allows B_12_ to achieve distal ileum where the complex B_12_-IF is finally absorbed by receptor-mediated endocytosis [85,86]. At this point, vitamin B_12_ exits through the basolateral membrane of enterocytes and binds to transcobalamin, its blood carrier, which delivers the vitamin to the cells.

A large amount of vitamin B_12_ is stored in the liver, while a smaller amount is excreted in the bile, taking part in enterohepatic circulation [85,86,87]. AAG may lead to irreversible damage of gastric oxyntic glands, leading to impaired gastric acid secretion and concomitantly reduced secretion of intrinsic factor. This, when the time goes by, may lead initially to subclinical vitamin B_12_ deficiency without anemia, and generally after a decade to overt PA [3,13].

### 3.3. Iron, the often Forgotten Missing Factor in Pernicious Anemia

Dietary iron is available as heme and non-heme iron [88]. Heme iron is contained in meat, it represents about 5–10% of the total dietary iron in the Western diet, and is absorbed by the small intestine mucosal cells by means of a surface receptor. Non-heme iron is contained in vegetables, legumes, cereals, and fruits, representing 80% of dietary iron [89]. This latter form is transported through the apical membrane of the enterocyte by divalent metal-ion transporter 1 (DMT1) and into the circulation through ferroportin 1 (FPN1) [90]. Non-heme iron is not soluble and precipitates at pH 3, requiring for its absorption a reduction to the ferrous or chelated form [88]. In healthy conditions, HCl and ascorbic acid play a pivotal role in iron absorption, favoring the reduction of non-heme iron from its ferric form to the ferrous form. At acid pH (<3), ascorbic acid forms soluble chelates with ferric iron decreasing polymerization and precipitation [91]. Based on these mechanisms, some gastric disorders can impair iron absorption and consequent iron deficiency, or eventually, iron deficiency anemia.

AAG, the pathological gastric alteration in PA, characterized by atrophy of oxyntic glands and impaired gastric acid production, may lead to iron malabsorption and iron deficiency anemia [13]. Even if AAG has been often viewed as synonymous of PA, and therefore vitamin B_12_ malabsorption, iron absorption was corrected by giving gastric juice to PA patients as early in the 1960s [92,93], linking HCl to the iron absorption process. Dickey et al. reported the presence of gastric corpus atrophy in about 20% of iron deficiency anemia patients diagnosed by gastric histology together with high serum gastrin levels [94]. Other authors showed corpus gastric atrophy, autoimmune or not autoimmune, in 19.5% to 26% of iron deficiency anemia patients without gastrointestinal symptoms [95,96]. Hershko et al., observed that 27% of iron deficiency anemia patients without evident gastrointestinal disorders had AAG [97]. More recently, iron deficiency and iron-deficiency anemia were found in 34% and 13.1% of AAG patients, respectively [79]. Thus, in patients with iron deficiency or iron deficiency anemia without manifest or occult bleeding, the presence of AAG needs to be ruled out [98,99]. Patients with AAG and iron deficiency anemia may over time present subclinical and later clinical PA due to the occurrence of IF and vitamin B_12_ deficiency, as sometimes iron deficiency may precede the development of PA [97]. In a previous study, normocytic MCV was found at presentation in 48 (30%) of AAG patients, of whom 24 (50%) had concomitant iron deficiency indicated by abnormal transferrin saturation and serum ferritin concentration; further, stratification by age from under 20 years to over 60 years of age showed a regular and progressive increase in MCV and serum ferritin levels, but a decrease in cobalamin levels [97]. Taking into consideration these data, it is advisable to proactively search for iron deficiency by assessing serum levels of iron, transferrin and ferritin in patients with PA, in particular when the typical macrocytosis (MCV > 100 fL) is not present. Likewise, it is advisable to monitor for the occurrence of subclinical cobalamin deficiency in patients with AAG by assessing serum vitamin B_12_, homocysteine, and folate levels.

### 3.4. Calcium, Vitamin D, and Ascorbic Acid, the Concealed Factors in Pernicious Anemia

Calcium absorption begins as a result of the intragastric acid pH promoting the dissolution of calcium salts to form soluble calcium chloride; this step facilitates proper calcium absorption in the proximal small intestine [100,101]. Several studies have been directed to clarify the match between PA and calcium malabsorption. Despite some controversies, a significant loss of bone mineral density in postmenopausal women affected by PA was reported. Furthermore, in AAG, the prevalence of osteopenia and osteoporosis is currently not known. Probably, this may be due also to the unavoidable bias of postmenopausal women, in whom both PA and osteoporosis or osteopenia are frequent.

Concerning calcium absorption, the role of vitamin D should be considered [102]. Significantly lower 25-OH vitamin D levels in AAG patients than in those with non-autoimmune atrophic gastritis, or in the general population, were reported, supporting the idea that low levels of vitamin D might be a risk factor for the development of autoimmune conditions [103]. Further, in AAG patients, an increased prevalence of hyperparathyroidism secondary to vitamin D deficiency was shown [104], even if the postmenopausal status was unknown.

Another forgotten micronutrient in PA is ascorbic acid. The most important determinant of plasma ascorbic acid levels in humans is diet, because humans are not able to synthesize it due to the loss of gluconolactone oxidase, necessary for the last step of vitamin C biosynthesis. After absorption, vitamin C is actively secreted and concentrated in the gastric juice in its reduced form of ascorbic acid, resulting in higher levels in the gastric juice than in the plasma [105]. Ascorbic acid is a crucial promoter of iron absorption, and when its bioavailability is reduced this may impair iron absorption. Analogously to the intrinsic factor necessary for vitamin B_12_ absorption, the “intrinsic factor” ascorbic acid is required for iron absorption [106]; ascorbic acid converts the ferric iron to its ferrous form that remains soluble in the alkaline duodenal environment and constitutes chelates with ferric chloride stable at a pH > 3 [107]. *H. pylori* infection was related to reduced vitamin C levels in the gastric juice and plasma [108,109,110,111]. One proposed mechanism was that lower bioavailability, insufficient vitamin C intake, hypochlorhydria, higher consumption due to increased active secretion from plasma to gastric juice in an attempt to restore the positive juice/plasma ratio, and *H. pylori*-associated oxidants accelerate ascorbic acid degradation [112]. In PA, in which hypochlorhydria is virtually always present, the prevalence of ascorbic acid deficiency is not known [3]. The possible metabolic relationship between ascorbic acid and vitamin B_12_ was assessed, showing lower plasma ascorbate levels before cobalamin supplementation that increased after vitamin B_12_ treatment [113]. The occurrence and eventual clinical implications of ascorbate deficiency in individuals with PA still need awaits definite clarification.

**Figure 1:**
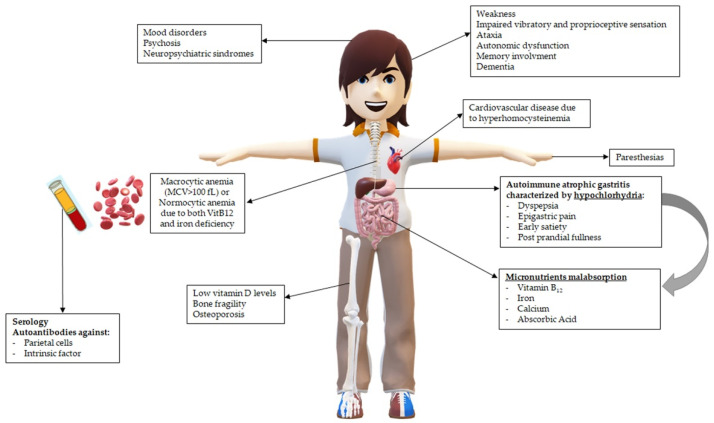
Main clinical consequences in patients with pernicious anemia (PA) related to hypochlorhydria and vitamin B_12_ deficiency.

The main clinical consequences in patients with PA related to hypochlorhydria and vitamin B12 deficiency are pictured in Figure 1.

## 4. Diagnosis of Pernicious Anemia: A Smart Combination of Biochemistry and High-Quality Endoscopy and Histopathology

As reported above, clinicians should raise the clinical suspicion for PA in many clinical scenarios, ranging from anemia and dyspepsia to autoimmune comorbidities and neurological and psychiatric alterations, as well as particular situations linked to vitamin B_12_ deficiency such as infertility or miscarriage. Based on the level of clinical suspicion, a step-by-step approach can be performed, starting with noninvasive tests that, when positive, will indicate gastroscopy with biopsies. When clinical suspicion is very high, the patient can directly undergo gastroscopy with biopsies to diagnose the presence of AAG and the consequent diagnosis of PA.

### 4.1. Biochemical Tests: The Non-Invasive Approach

The mainstream manifestation of PA is macrocytic anemia referring to macrocytosis (mean corpuscular volume (MCV) greater than 100 fL) in the setting of anemia (hemoglobin less than 12 g/dL in non-pregnant females, hemoglobin less than 11 g/dL in pregnant females, or hemoglobin less than 13 g/dL in males) [114]. The greater MCV, observed in the setting of PA, is intrinsically connected to B_12_ deficiency, resulting from disruption of DNA synthesis. Cobalamin deficiency leads to H4-folate synthesis impairment, limiting the availability of the required form of folate for thymidylate and DNA synthesis. This is the reason for the misincorporation of dUTP instead of thymidine triphosphate during DNA synthesis [87,115]. Such unbalanced development in dividing bone marrow cells leads to production of large cells with fine and immature-looking nuclear chromatin. Erythroid precursors are particularly affected, leading to anemia with large red cells. Furthermore, other hematopoietic cells are also affected: gigantic granulocyte precursors in the marrow and hypersegmented neutrophils in the blood. Finally, the whole marrow precursors may be impaired, eventually leading to pancytopenia [11]. The second essential condition in PA is cobalamin deficiency. Serum B_12_ levels above 300 pg/mL are considered as normal. Serum B_12_ levels between 200 and 300 pg/mL are interpreted as borderline, and further testing may be useful, as reported below. Serum B_12_ levels below 200 pg/mL are considered deficient [12].

In patients with borderline B_12_ levels (200 to 300 pg/mL), further enzymatic testing is indicated. The common consequence of cobalamin deficiency results in disorders affecting vitamin B_12_ metabolism, in particular, the cellular deficit in one or both of the coenzyme forms of B_12_, adenosyl-B_12_ and methyl-B_12_. Cobalamin impairment leads to a deficit in methylation and impaired metabolism of methylmalonate, derived from amino acids and fatty acids’ catabolism [11]. Methyl-B_12_ deficiency leads to homocysteine accumulation and reduced synthesis of methionine and S-adenosylmethionine. Hyperhomocysteinemia increases the risk of atherothrombosis, neuropsychiatric manifestations, such as paraesthesia, weakness, gait abnormalities, and cognitive or behavioral changes [116]. Adenosil-B_12_ deficiency results in the accumulation of methylmalonic acid (MMA). Homocysteine and MMA accumulations in plasma denote B_12_ deficiency, but homocysteine can be influenced by other parameters (folate and B_6_ deficiency, kidney and thyroid function, sex, and age) [11]. The most specific and sensitive single test for B_12_ deficiency is MMA, which is often used as gold standard for defining B_12_ status [117,118].

The biochemical assessment of patients with the diagnosis of PA should include laboratory tests on gastric autoantibodies useful for defining AAG diagnosis. The presence of PCA may be assayed by immunofluorescence, still used in many diagnostic immunology laboratories. Identification of H^+^/K^+^ ATPase as the target autoantigen has guided the development of an enzyme-linked immunosorbent assay (ELISA) for the detection of PCA [119]. A cross-sectional study on 516 adult patients with histologically proven AAG reported that about 20% of patients are seronegative at the time of AAG histological diagnosis, and this is more common in elderly individuals [120]. Moreover, a study suggested that testing for pepsinogen I, pepsinogen II, pepsinogen I to pepsinogen II ratio, and gastrin-17 may diagnose any type of atrophic gastritis [121]. These serological tests are known as serological gastric biopsy. Anti-*H. pylori* antibodies may help to identify patients who had a previous or have a current *H. pylori* infection. A combination of pepsinogens (pepsinogen I and pepsinogen II), gastrin-17, and anti-*H. pylori* antibodies serum assays seem to have a slightly higher diagnostic performance than serum pepsinogens and gastrin-17 tests alone [3]. A 2017 meta-analysis including 4241 atrophic gastritis patients to assess the diagnostic performance of such tests, reported above, showed a pooled sensitivity of 74.7% (95%CI 62–84.3) and a pooled specificity of 95.6% (95%CI 92.6–97.4) [122]. A recent paper showed that autoantibodies against the ATP4A or ATP4B subunits (assessed by luminescent immunoprecipitation system) and pepsinogen I were reliable serological pre-endoscopic markers in patients with corpus atrophic gastritis; in particular, ATP4B, ATP4A, and pepsinogen I tests showed sensitivities of 77%, 75%, and 73% and specificities of 88%, 88%, and 80%, respectively, with the ATP4B test showing the highest diagnostic performance (*p* = 0.008 vs. ATP4; *p* = 0.0002 vs. pepsinogen I) [123]. IFA are considered serological markers of PA, highly specific but with low sensitivity [3,31], and have gained increased importance because the Schilling test has become obsolete. In chronic atrophic gastritis patients, a recent paper using LIPS assay showed sensitivity and specificity of 32% and 95% of IFA, and this diagnostic performance was similar in patients with or without vitamin B_12_ deficiency or anemia, suggesting that positivity against IFA may indicate latent vitamin B_12_ deficiency [124]. Unfortunately, the IFA assays are not widely available.

### 4.2. Gastroscopy and Histopathological Assessment: The Required Confirmation of the Diagnosis of Pernicious Anemia

Patients with diagnosis of PA should undergo gastroscopy with biopsies to ascertain the presence of AAG, keeping in mind that PA patients are at increased gastric cancer risk. Unfortunately, this fearsome complication may be found sometimes at the time of diagnosis of PA, as PA is a late-stage clinical manifestation of AAG.

In Western countries, biopsies and histological confirmation of the presence of atrophy are considered mandatory for the diagnosis of gastric atrophy and thus PA. Otherwise, in Eastern countries, the diagnosis of atrophic gastritis is based on the endoscopic Kimura–Takemoto classification [125]. thus waiving gastric biopsies which are performed only when intestinal metaplasia is suspected. However, in Western countries, this classification is less used because of its low interobserver reliability, and also due to the presence of AAG, where the corpus atrophic damage makes such classification not applicable. For this reason, biopsies are obtained to stage the presence of atrophic gastritis and the eventual presence of intestinal metaplasia in the stomach using OLGA/OLGIM classification [126,127]. Biopsies should be collected following the updated Sydney system [20]: two biopsies of the antrum and two biopsies from the corpus should be obtained and sent in separate vials. Another biopsy should be performed from *incisura angularis* and sent in the same vial of antrum biopsies.

Electronic chromoendoscopy, defined as the application of a specific source of light during endoscopy, allows us to recognize intestinal metaplasia, therefore it would be considered a valuable tool in the hand of clinicians. Cumulative evidence suggests that electronic chromoendoscopy is highly accurate for the diagnosis of precancerous conditions [128,129,130]. A recent trial demonstrated that Narrow-Band Imaging (NBI), a specific type of electronic chromoendoscopy, is superior to standard white-light endoscopy in identifying patients with intestinal metaplasia [131], showing that NBI is useful for the diagnosis of intestinal metaplasia. Moreover, NBI reliably identifies intestinal metaplasia with accuracy rates of about 85–90% [20] in the general population undergoing gastroscopy for upper gastrointestinal symptoms or screening of gastric cancer, thus opening up the possibility to limit and even waive gastric biopsies in the absence of intestinal metaplasia, saving time and money [132]. Recently, a new type of electronic chromoendoscopy, namely Blue-Light Imaging (BLI) was developed, and showed a great accuracy for the presence of intestinal metaplasia [133]. A new classification, namely endoscopic grading of gastric intestinal metaplasia (EGGIM) has been proposed and validated to score endoscopic assessment of intestinal metaplasia with the use of high-resolution (HR)-NBI gastroscopes [134]. EGGIM showed high accuracy in the staging of intestinal metaplasia compared to histological assessment as the gold standard, showing sensitivity and specificity of 89.4 and 94.6% in the diagnosis of extensive intestinal metaplasia (OLGIM III/IV), respectively. In a cross-sectional study on 210 AAG patients, EGGIM confirmed a good accuracy and a high sensitivity, reliably identifying more than 90% of patients with histological corpus IM. However, an overestimation of intestinal metaplasia was reported when pseudopyloric metaplasia was present [135]. The metaplastic transformation of the gastric epithelium because of chronic inflammation may give rise to two types of metaplasia: intestinal metaplasia and pseudopyloric metaplasia. Intestinal metaplasia is part of the Correa Cascade as a well-known precancerous condition [136]. Pseudopyloric metaplasia represents the replacement of the oxyntic mucosa by antral-type mucosa, according to a phenomenon known as antralisation [137]. A recent study conducted on a longitudinal cohort of 292 patients with corpus atrophic gastritis, adhering to endoscopic-histological surveillance, showed that at 4.2 years of follow-up, the only presence of pseudopyloric metaplasia, without the concomitant presence of intestinal metaplasia, was not associated with the development of gastric dysplastic lesions or gastric cancer, because these lesions were consistently associated with corpus intestinal metaplasia [138]. These findings emphasize that corpus intestinal metaplasia can be viewed as a cornerstone in gastric carcinogenesis, as reported in Correa Cascade and subsequently supported by data [139,140,141].

**Figure 2:**
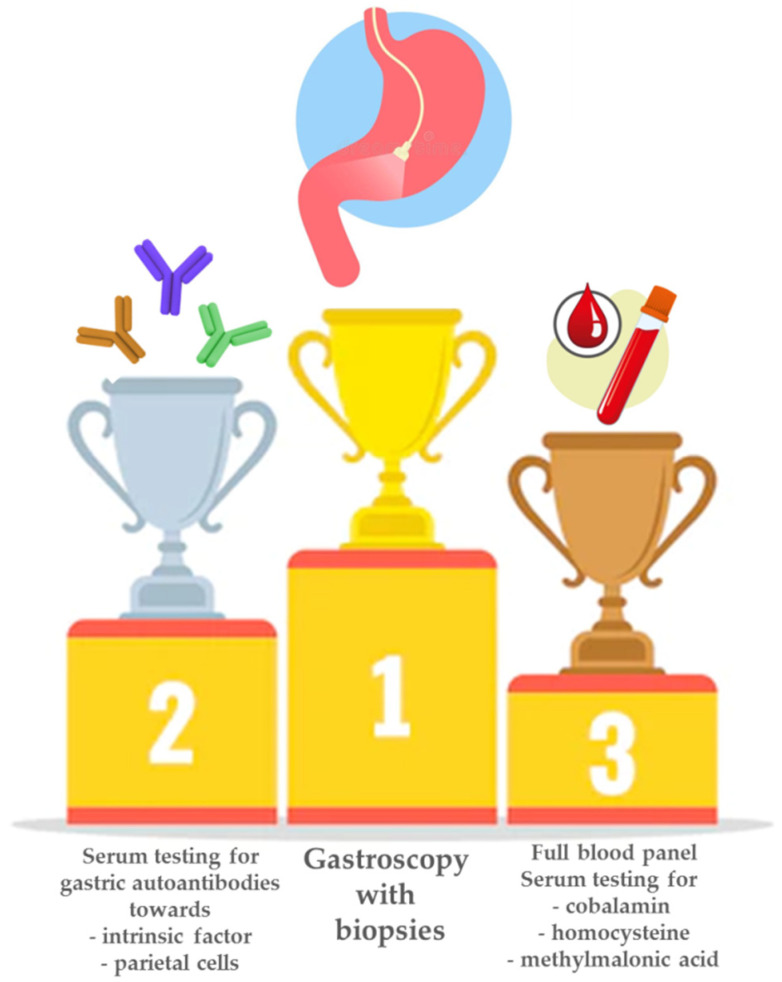
Mainstays in the diagnosis of pernicious anemia. When pernicious anemia is suspected, the first step is usually a full blood panel to test for anemia and/or macrocytosis, together with testing for cobalamin deficiency and increased levels of homocysteine and/or methylmalonic acid. Next, the positivity of gastric autoantibodies towards parietal cells and/or intrinsic factor is commonly assessed. In any case, the hematological and/or serological suspicion of pernicious anemia always needs to be confirmed by histological assessment of gastric antral and corpus biopsies obtained during gastroscopy.

Thus, for a correct diagnosis of PA and an optimal risk stratification for the possible future development of gastric neoplastic lesions, high-quality endoscopy with correctly applied biopsy protocols is needed. Figure 2 shows a schematic of the mainstays of diagnosis of PA.

## 5. Long-Term Complications and Clinical Management of Pernicious Anemia: Good Protocols May Reduce Burden and Long-Term Risks

As described in the previous paragraphs, PA is a complex disease. For this reason, complications can involve different aspects of the patient, impairing the quality of life.

### 5.1. Long-Term Risks in Patients with Pernicious Anemia from a Hematological and Gastroenterological Point of View

From a hematological point of view, the most common long-term complications in patients with unrecognized and/or untreated PA are neurological manifestations due to the demyelination of peripheral and central neurons [11,87] as a consequence of vitamin B_12_ deficiency; unfortunately, once treated with vitamin B_12_ supplementation, these alterations are not always reversible. Other, harmful and potentially life-threatening complications in untreated or unmonitored PA patients are related to anemia due to iron and/or cobalamin deficiency, which may be severe, with a usually slow and underhand onset, possibly leading to acute cardiovascular complications (acute heart attack), and the risk of thrombosis related to hyperhomocysteinemia, possibly leading to stroke, occlusive artery disease, or venous thrombosis [3].

From a gastroenterological point of view, the most severe long-term risk in patients with PA is gastric cancer. In a systematic review in 2013 [10], a 6.8-fold relative risk for gastric cancer and an overall pooled gastric cancer incidence rate per person-years of 0.26% with a range between 0% and 1.2% was shown. However, studies have presented a high heterogeneity of study populations and design, and the risk of progression varied between studies, as confirmed in a recent systematic review [142], where the risk of progression of chronic atrophic gastritis to gastric adenocarcinoma ranged from 0.1–0.3% per year [143,144,145]. Patients with chronic atrophic gastritis are also at high risk of developing type 1 neuroendocrine tumors [146], with an incidence rate of about 0.4–0.7% per year with heterogeneity between studies [147,148], in particular in patients with PA [149].

Concerning the risk for other types of cancer, excluding gastric cancer, a meta-analysis [150] showed that PA presented a cumulative incidence of 2.4% for a cumulative follow-up of 1855 years, with a higher risk than the general population for biliary tract cancer (1.81: 1.21–2.70) and hematological malignancies such as multiple myeloma (2.83: 1.76–4.55), Hodgkin’s lymphoma (3.0: 1.35–6.68), non-Hodgkin’s lymphoma (2.08: 1.58–2.75), and leukemia (1.56: 1.16–2.12).

### 5.2. Supplementation and Clinical Follow-Up: Always Be Alert

Due to micronutrient deficiencies, the main therapy of PA is represented by the micronutrient supplementation [3]. Concerning cobalamin supplementation, oral administration and intramuscular administration could be both prescribed, as a Cochrane Review of 2018 showed the same results in restoring cobalamin deficiency [151]. However, intramuscular administration should be preferred in some scenarios: at diagnosis, to quickly restore the deficiency, especially if the patient presents neurological symptoms, or during the follow-up to restore a very low level of cobalamin. Oral administration of cobalamin has the advantages of easier administration at lower costs. However, the evidence is scarce on this topic, and cobalamin administration should be individualized.

Concerning iron deficiency, supplementation should be considered in patients with severe deficiency. Intravenous supplementation could quickly restore the deficiency, but oral administration is sometimes preferred as evidence of the superiority of intravenous versus oral administration is scarce [152], and specific data on PA on this topic are lacking. The choice for one treatment rather than another could be based on the severity of deficiency and side effects. The choice of oral iron administration depends mostly on the fear of allergic reactions to intravenous iron, even if gastrointestinal symptoms such as abdominal pain, dyspepsia and diarrhea, could occur for oral iron administration. However, in a meta-analysis conducted by Avni et al. [153] on the efficacy and safety of intravenous iron preparations, the occurrence of severe adverse events and other adverse events were not increased in intravenous iron preparations compared to controls. Overall, 103 studies were considered. Concerning severe adverse events, 97 studies were included and reported that intravenous iron preparations did not show an increase in the risk of serious adverse events compared to controls (RR 1.04). Furthermore, no increase in mortality (RR 1.06) or other adverse events (RR 1.04) was found. Based on the efficacy of intravenous iron preparations in terms of rapidity in restoring iron deficiency anemia, and the safety of intravenous iron formulations, the first choice of iron supplementation especially in severe iron deficiency anemia should be intravenous supplementation. The clinical follow-up of patients with PA is usually scheduled once a year to detect long-term consequences of PA, in particular to assess the presence of anemia or the clinical effect of supplementations, but also to evaluate the presence of digestive symptoms such as dyspepsia or epigastric pain. Alarming symptoms, namely the onset of iron deficiency anemia or loss of body weight, but also a recent onset of troublesome new dyspeptic symptoms, could require an immediate endoscopic evaluation.

### 5.3. Endoscopic Follow-Up: New Techniques to Increase the Diagnostic Yield of High-Risk Lesions

Due to the risk of developing gastric cancer and type 1 gastric neuroendocrine tumors, endoscopic surveillance is the mainstay of the follow-up in PA, as the early detection of neoplastic lesions at follow-up could permit a curative resection. European guidelines [140] suggest a 3-year follow-up in patients with metaplastic atrophic gastritis only in the antrum or only in the corpus, if risk factors, namely autoimmune gastritis, first-degree family history of gastric cancer, incomplete intestinal metaplasia, or persistent *H. pylori* infection, are present. In case of atrophic gastritis or intestinal metaplasia involving both antrum and corpus, a 3-year follow-up is recommended unless a first-degree family history is present. In this case, endoscopy should be performed every 1–2 years. The 3-year follow-up was shown to be cost-effective in intermediate-risk gastric cancer countries [154] and was confirmed in another Italian cost-effective study [155]. However, only a few studies were performed to investigate the best interval to follow-up patients harboring this condition. In two studies conducted proposing a 2-year interval, the development of neoplastic lesions was present earlier than the 3-year interval [156,157]. However, other studies showed that even in patients with non-extensive atrophic gastritis, namely OLGA I-II/OLGIM I-II, neoplastic lesions could develop [147,158,159,160]. Furthermore, a recent longitudinal cohort study, conducted on 122 patients with AAG and 38 patients with multifocal atrophic gastritis in a follow-up program with a 3-year interval, showed the presence of 16 gastric neoplastic lesions at follow-up: 18.7% gastric cancers, 25.0% low-grade dysplasia, 12.5% low-grade dysplasia adenomas and 43.7% type-1 neuroendocrine tumors. All the diagnosed lesions were endoscopically (87.5%) or surgically (12.5%) treated with favorable outcomes [161].

The management of type 1 neuroendocrine tumors and resection strategy have still not been clarified [162]. The European Neuroendocrine Tumor Society guidelines suggest removing tumors larger than 10 mm as they represent neuroendocrine tumors with potentially more aggressive behavior [163]. In a recent prospective study conducted on 80 patients presenting 127 gastric type 1 neuroendocrine tumors, the authors concluded that resection strategy could be planned based on the tumor size. Neuroendocrine tumors smaller than 5 mm could be surveilled, and could be removed by cold snare polypectomy when the size was between 5 and 10 mm and by EMR or ESD when bigger than 10 mm, even if with low evidence [164].

European guidelines suggest that surveillance in patients with gastric precancerous conditions should be performed with high-definition electronic chromoendoscopy to better recognize areas at risk for gastric cancer and to perform gastric biopsies. Three meta-analyses showed the high accuracy of conventional and virtual chromoendoscopy for the diagnosis of early gastric cancer and dysplasia in patients harboring precancerous conditions [129,165,166]. Moreover, in pilot studies, virtual chromoendoscopy seemed to be useful for the characterization of type-1 neuroendocrine tumors [167].

In this setting, the use of artificial intelligence seems to be a promising tool to optimize the diagnosis of precancerous and cancerous lesions of the stomach and to increase the diagnostic yield of high-risk lesions [168,169].

## 6. Conclusive Remarks

In conclusion, this heterogeneous and complex disorder merits increased attention from clinicians of different medical specialties, and common efforts should be made to increase awareness of clinical red flags that should immediately raise the clinical suspicion of PA. This is very important, as a delayed diagnosis may lead to serious consequences, such as untimely corrected micronutrient deficiencies, in particular of vitamin B_12_ and iron, that may lead to potentially serious complications such as heart, vascular or neurological conditions, which sometimes remain irreversible despite supplementation.

The correct diagnosis of PA should always be confirmed by high-quality gastroscopy with biopsies. This is important in order to confirm or rule out the presence of AAG, but it is also important to rule out the presence of neoplastic complications, such as gastric dysplasia, gastric cancer or type 1 neuroendocrine tumors, which sometimes may already be present at the time of diagnosis of PA, which is the end-stage clinical manifestation of AAG.

At least yearly scheduled clinical and biochemical monitoring is essential to diagnose new micronutrient deficiencies in a timely manner, and regular endoscopic surveillance is necessary for early detection of gastric neoplastic complications.

The optimal clinical management of PA requires continuous crosstalk and a practical translationality between several medical specialties, so as to not lose the global sight of this complex disease and to guarantee the best possible care and outcome to the patient.

## Figures and Tables

**Table 1:** Prevalence, age and gender in pernicious anemia.

		References
Prevalence		
General population	0.1%	[6,8,11]
Over 65 years	2–3%	
Average age		
-Female	40–61 years	[5]
-Male	49–50 years	
Female/male ratio	2:1	[7,8,11]

## Data Availability

Not applicable.
